# Performance evaluation for rapid detection of pan-cancer microsatellite instability with MANTIS

**DOI:** 10.18632/oncotarget.13918

**Published:** 2016-12-12

**Authors:** Esko A. Kautto, Russell Bonneville, Jharna Miya, Lianbo Yu, Melanie A. Krook, Julie W. Reeser, Sameek Roychowdhury

**Affiliations:** ^1^ Comprehensive Cancer Center, The Ohio State University, Columbus, OH, USA; ^2^ Department of Biomedical Informatics, The Ohio State University, Columbus, OH, USA; ^3^ Division of Medical Oncology, Department of Internal Medicine, The Ohio State University, Columbus, OH, USA

**Keywords:** microsatellite instability, computational biology, next-generation sequencing

## Abstract

In current clinical practice, microsatellite instability (MSI) and mismatch repair deficiency detection is performed with MSI-PCR and immunohistochemistry. Recent research has produced several computational tools for MSI detection with next-generation sequencing (NGS) data; however a comprehensive analysis of computational methods has not yet been performed. In this study, we introduce a new MSI detection tool, MANTIS, and demonstrate its favorable performance compared to the previously published tools mSINGS and MSISensor. We evaluated 458 normal-tumor sample pairs across six cancer subtypes, testing classification performance on variable numbers of target loci ranging from 10 to 2539. All three computational methods were found to be accurate, with MANTIS exhibiting the highest accuracy with 98.91% of samples from all six diseases classified correctly. MANTIS displayed superior performance among the three tools, having the highest overall sensitivity (MANTIS 97.18%, MSISensor 96.48%, mSINGS 76.06%) and specificity (MANTIS 99.68%, mSINGS 99.68%, MSISensor 98.73%) across six cancer types, even with loci panels of varying size. Additionally, MANTIS also had the lowest resource consumption (<1% of the space and <7% of the memory required by mSINGS) and fastest running times (49.6% and 8.7% of the running times of MSISensor and mSINGS, respectively). This study highlights the potential utility of MANTIS in classifying samples by MSI-status, allowing its incorporation into existing NGS pipelines.

## INTRODUCTION

Microsatellites are short (1-6bp) repeating motifs, widely dispersed throughout the human genome [1]. Microsatellite instability (MSI) is a genetic phenomenon of somatic polymorphisms of microsatellite length, caused by uncorrected “slippage” of DNA fragments during DNA replication in cell division. MSI can arise from defects in the DNA mismatch repair (MMR) system [2]. These defects may be inherited, as with Lynch syndrome/hereditary nonpolyposis colorectal cancer [3], or may be somatically acquired, most commonly due to promoter hypermethylation of the MMR gene *MLH1* [4]. Increasing evidence demonstrates that MSI is a recurrent somatic abnormality in several human cancers, found in 13% of colorectal adenocarcinoma and 22-33% of uterine/endometrial carcinoma [5]. Reliable detection of MSI is clinically useful as MSI-positive tumors appear more susceptible to immune-enhancing therapies, as observed in colorectal cancer for the PD-1 inhibitor pembrolizumab [6].

The two currently accepted assays for the detection of MSI are MSI-PCR of five standardized microsatellite loci (Bethesda panel) [7], and immunohistochemistry (IHC) of the MMR proteins MSH2, MSH6, MLH1 and PMS2. Traditionally, tumors can be classified with MSI-PCR as microsatellite stable (MSS, 0/5 loci unstable), MSI-low (MSI-L, 1/5 loci unstable), or MSI-high (MSI-H, ≥ 2/5 loci unstable) [7]. However, both of these methods have inherent limitations. MSI-PCR relies on a small set of loci that were selected based on markers from a single disease type, potentially excluding loci that would be better predictors in other diseases and increasing the odds of incorrect classification [7]. Immunohistochemistry can be used to detect the expression of mismatch repair proteins, but does not directly look at the microsatellite loci [7]. More recently, with the increasing prevalence of next-generation sequencing (NGS) in cancer biology, several computational methods have been developed using either colorectal or endometrial cancer NGS data to determine MSI status [8, 9].

The development, refinement, and validation of NGS-based computational MSI calling methods have several research and clinical applications. NGS allows for the practical assessment of far more microsatellite loci than MSI-PCR. Importantly, computational MSI analyses can be integrated into existing NGS pipelines for other mutation types such as single nucleotide variation or copy number variation, as well as applied to previously generated NGS data for retrospective analyses. As NGS increases in both cost-effectiveness and prevalence, NGS-based methods may permit identification of MSI status without requiring additional clinical testing or patient sample processing. Lastly, NGS data is becoming increasingly available in tumor types that are not routinely tested for MSI, with potential opportunities to identify microsatellite instability in previously uncharacterized cancers. Thus, there is a need to develop tools with high accuracy in multiple cancer types.

In this study, we introduce a new tool, MANTIS (Microsatellite Analysis for Normal Tumor InStability), for detecting MSI status from NGS data. We compare MANTIS with two currently available tools, mSINGS [8] and MSISensor [9], and test their performance across six different tumor types. We also determine that the number of loci assessed impacts the accuracy of MSI calling methods, and find an optimal number of loci to analyze with each tool for best tool performance.

## RESULTS

### Evaluation of tool accuracy for detecting MSI status

Since mSINGS and MSISensor were each developed on data from single disease cohorts (COAD/READ for mSINGS, UCEC for MSISensor), the two cohorts were used for tool performance comparisons. To account for the possibility of suboptimal recommended cutoff thresholds for each of the three tools, we tested a range of thresholds for each tool across a test cohort consisting of both the COAD/READ and UCEC sample pairs (Supplemental Figure S1). The peak performances of each tool were determined, with MANTIS having 97.1% accuracy with the default threshold of 0.4, mSINGS reaching 96% accuracy with a threshold of 0.1, and MSISensor peaking at 95.4% accuracy with the threshold of 3.5%. The results indicate that MANTIS demonstrated superior performance over the other tools, even after accounting for the best-case thresholds of the tools.

Having evaluated the tools with the best-case thresholds, their performances were evaluated with the tool's recommended default cutoff thresholds (MANTIS 0.4, mSINGS 0.2, and MSISensor 3.5%) (Supplemental Figure S2). MANTIS demonstrated the highest classification accuracy (97.1%), followed by MSISensor (95.4%), and mSINGS (83.4%). MANTIS and MSISensor both exhibited equally high sensitivity (95.4%). In contrast, although mSINGS was the most specific (100%), it demonstrated poor sensitivity (66.7%), largely due to poor performance over the UCEC cohort (53.1% sensitivity). MANTIS also exhibited high specificity (98.9%), performing better than MSISensor (95.5%).

To analyze disease-specific differences, results were compared between the COAD/READ and UCEC cohorts (Table 3, Supplemental Figure S3). MANTIS produced more accurate results (98.7%) than mSINGS (92.1%) and MSISensor (92.1%) in COAD/READ, whereas MSISensor was slightly more accurate (98.0%) than MANTIS (96.0%) in UCEC. While all three tools performed well with the COAD/READ data, mSINGS performed poorly with the UCEC data (accuracy 76.8%, sensitivity 53.1%). The tests showed that MANTIS had the most consistently accurate performance among the test cohort, exhibiting high sensitivity (95.4%) and specificity (98.9%) among the tested samples.

### Consideration of data overfitting and bias

To further evaluate tool performance and to ensure MANTIS was not overfit to the COAD/READ and UCEC cohorts, tool performance was evaluated using stomach adenocarcinoma (STAD) sample pairs as a blinded test cohort (Figure 2). All three tools performed well with the STAD data, with MANTIS performing the best with 100% accuracy (Table 3), followed by MSISensor with 99% accuracy and mSINGS with 96% accuracy (Table 3).

**Figure 1:**
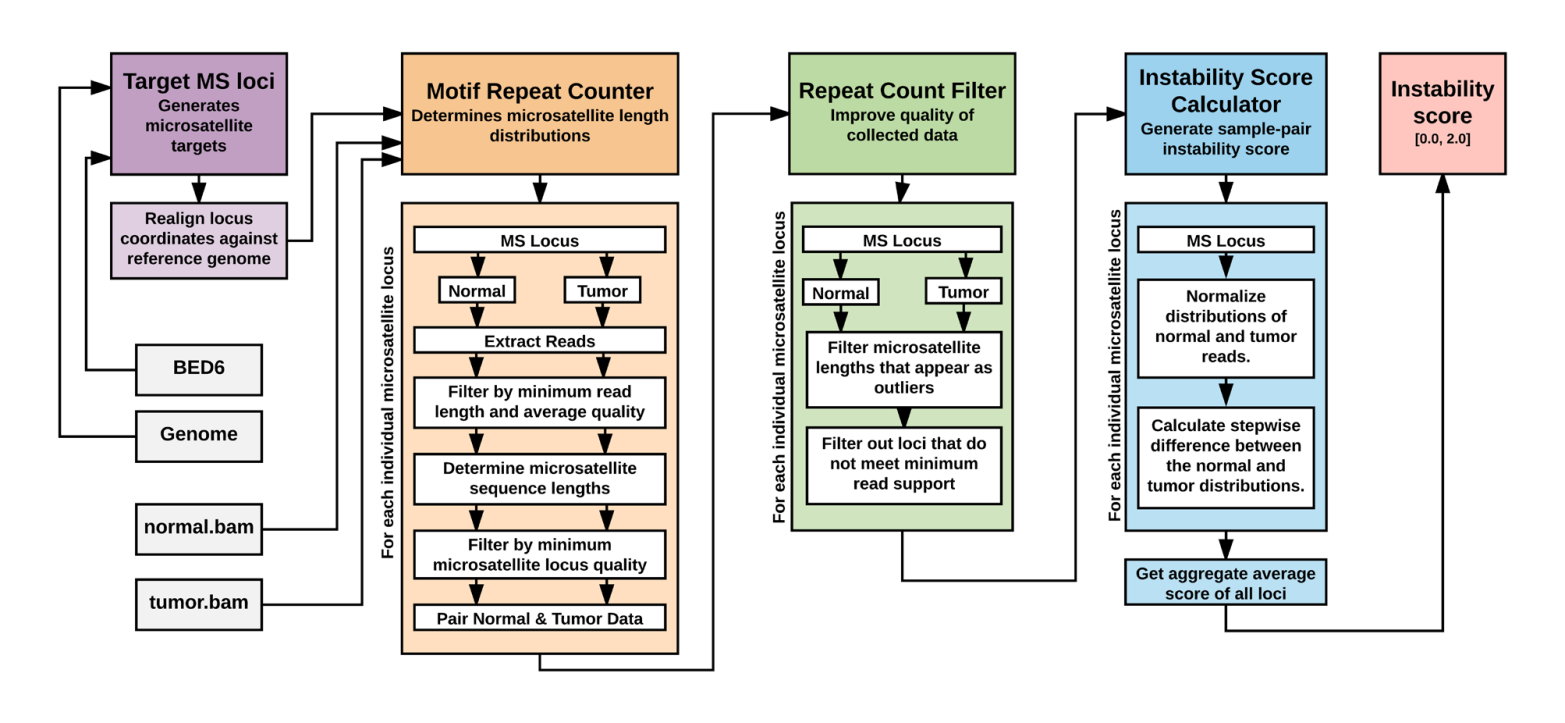
The schematic of the MANTIS analysis for MSI detection Microsatellite loci are realigned against the reference genome to account for 0/1-based indexing differences. Per-locus microsatellite length distributions are determined from the normal and tumor BAM files by extracting locus-spanning reads; filtering out reads that fail to meet minimum length and average base quality requirements; determining the start position of the microsatellite motif within each read's sequence and the number of motif repeats; ensuring such reads meet minimum average locus quality and aren't prematurely truncated in the middle of a motif repeat. The generated normal and tumor length distributions are evaluated at each locus, with outlying length values (by default, > 3 SD from mean) removed. Loci with substandard coverage are also removed. The support counts at each locus are then normalized separately for the normal and tumor sample and the stepwise difference between each distribution is calculated. Finally, the average of all difference scores is taken to generate the instability score for the normal-tumor sample pair.

**Figure 2:**
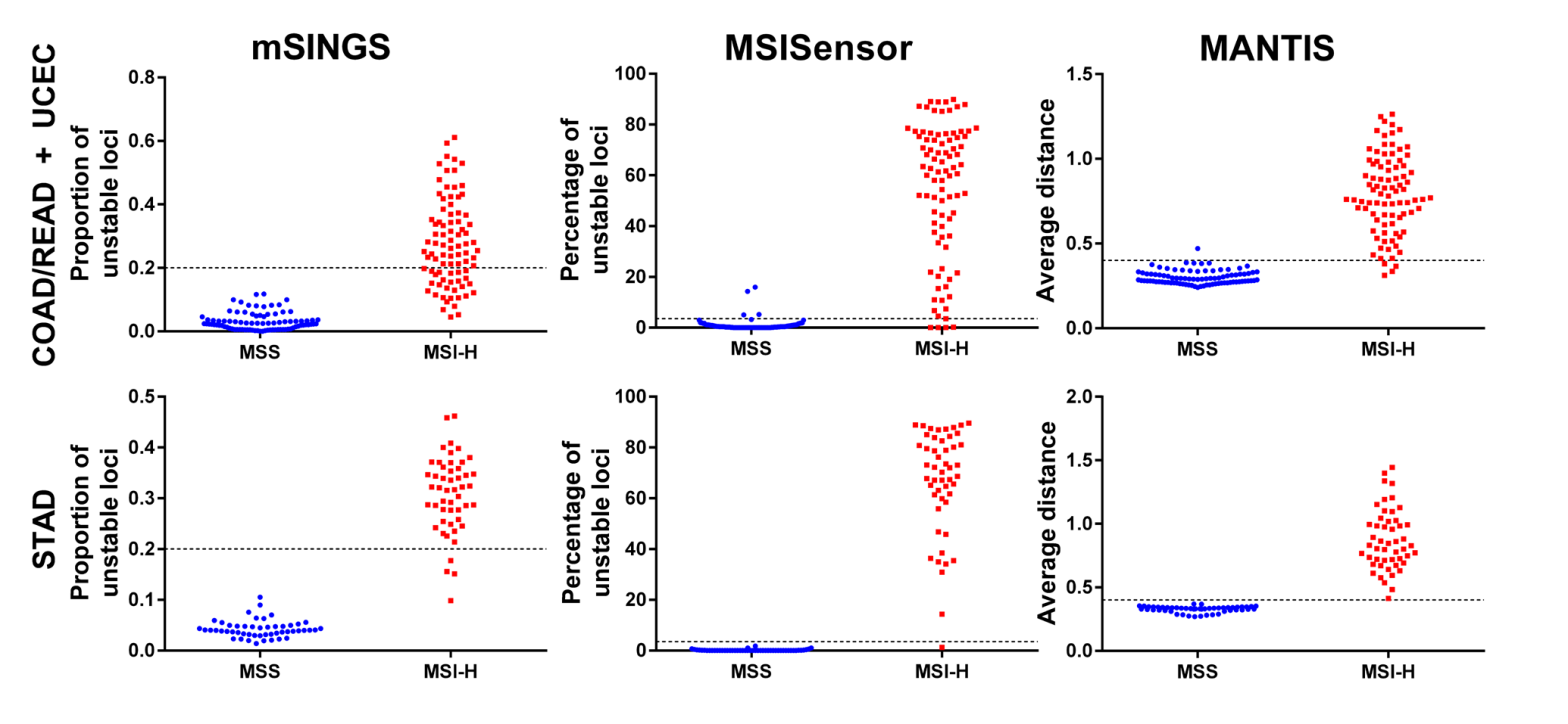
The cumulative distribution of MSI scores reported by mSINGS, MSISensor, and MANTIS for 275 COAD/READ, UCEC and STAD tumor-normal data pairs The dotted lines are the tools’ respective thresholds to call a tumor MSI positive.

To evaluate the extent that tool performance was affected by differences in sequencing, samples that had been sequenced at different sequencing centers with potentially different protocols and practices, were compared. For the comparison, we evaluated tool performance on a selection of MSI-H sample pairs that were each sequenced at two different centers (Supplemental Table S3). All three tools showed high concordance (R^2^ > 0.99 in all cases) for the 20 UCEC tumor-normal pairs used in these comparisons. For the 4 COAD/READ pairs, MSISensor and MANTIS showed high concordance (R^2^ > 0.99), however no concordance was observed with mSINGS (R^2^ = 5.52 · 10^-7^). This indicated that while MANTIS and MSISensor are less affected due to their paired normal-tumor comparison model, mSINGS requires a statistically large enough population to generate a baseline from.

Finally, to assess the potential utility of MANTIS as well as other tools for pan-cancer MSI analysis, we tested MANTIS, mSINGS, and MSISensor with three additional cohorts of cancer: esophageal carcinoma (ESCA), uterine carcinosarcoma (UCS), and prostate adenocarcinoma (PRAD) (Supplemental Table S4). All three tools reached 100% accuracy with ESCA, and MSISensor and MANTIS were 100% accurate with UCS and PRAD. However, mSINGS only reached 50% sensitivity (and 98.1% accuracy) with UCS data, and had one false positive in PRAD (98.3% specificity and 98.3% accuracy). Testing with these four additional disease cohorts further confirmed the accuracy of MANTIS, showing it may have superior pan-cancer applicability over the other two tools being compared.

### Computational performance

Since resource limitations can affect the rate at which computational analysis of samples can be performed, we used the tumor and normal samples of TCGA-V5-A7RE (a known ESCA MSS case) for evaluating the computational performance of the three tools, as both deduplicated BAM files were close to 10 GB in size. We found that mSINGS performs considerably slower than MSISensor and MANTIS, with runtime at least five-fold longer (Supplemental Table S2). mSINGS also requires substantially more memory and disk space than both MSISensor and MANTIS. This is because over 99% of the 32 GB of disk space used was temporarily occupied by the mpileup file created by mSINGS with SAMtools as an intermediate step. MANTIS exhibited a 20.5% speed increase when run using three threads *vs*. one thread, and MSISensor had a 12.8% slowdown when using three threads (Supplemental Table S2). The lack of expected three-fold performance scaling with either tool may be due to testing in an I/O-bound computing environment. The lower resource consumption and faster performance of both MANTIS and MSISensor indicated they may be better suited than mSINGS for a resource-limited environment, with MANTIS exhibiting the fastest runtimes.

### Accuracy of tools varies with both the number and specified loci evaluated

**Figure 3:**
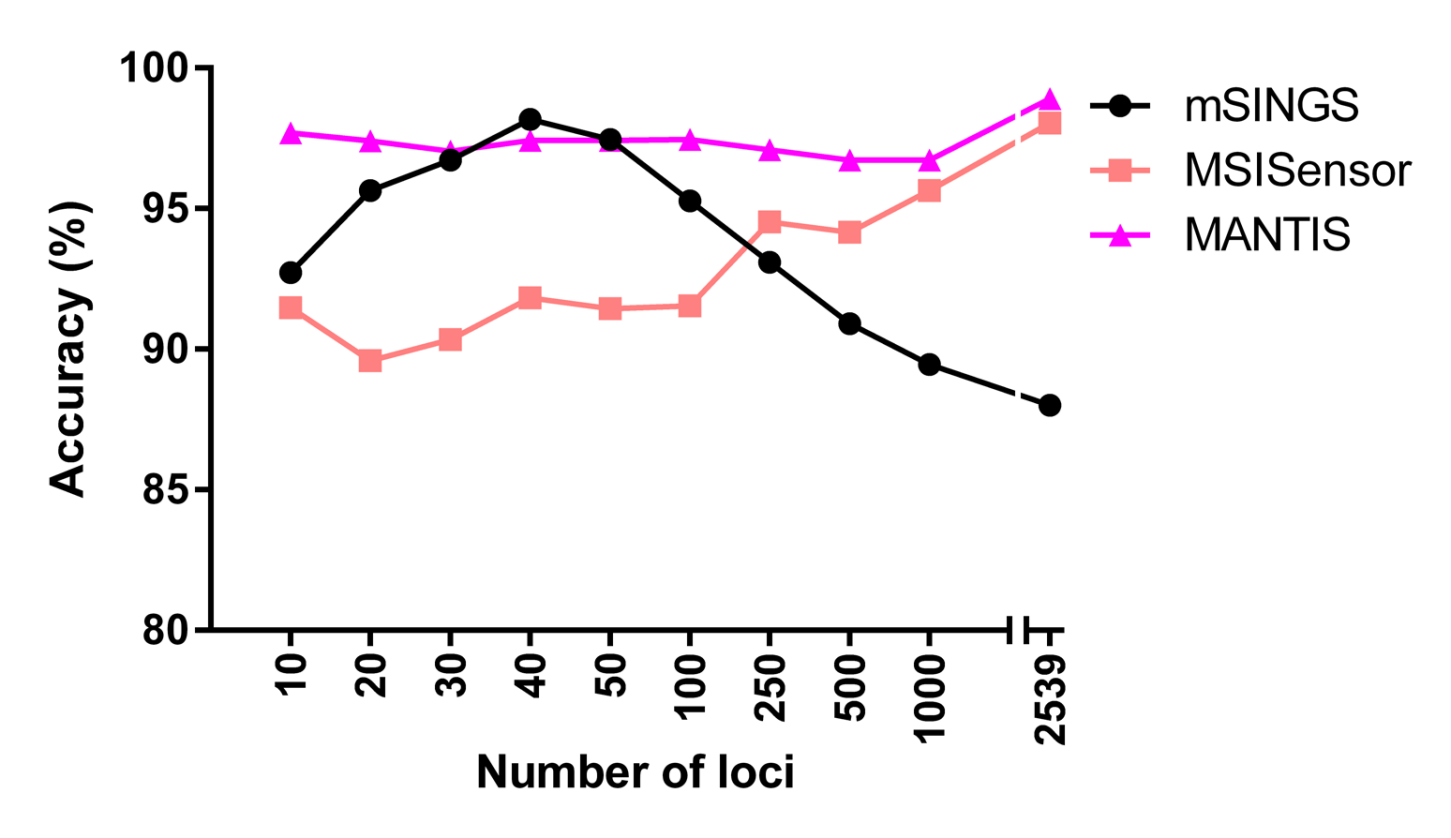
The performance of mSINGS, MSISensor and MANTIS with their respective top-performing loci The performance of each tool in each COAD/READ, UCEC and STAD cohorts was evaluated with top tool-specific loci (10-1000). The results with 2539 loci (without loci shortlisting) are included for reference.

To assess the effect of considering different numbers and selective microsatellite loci on MSI analysis, we identified the 10, 20, 30, 40, 50, 100, 250, 500 and 1000 loci most predictive of a sample's status across COAD/READ, UCEC and STAD cohorts, for mSINGS, MSISensor and MANTIS (Supplemental File S5). Each tool was then run with its list of top loci from all three cancer types (Figure 3, Supplemental Figure S4). Notably, with its top 40 loci, mSINGS was more accurate than MSISensor and MANTIS with their top 40 loci (98.2%, 91.8% and 97.4% accuracy, respectively) (Figure 3). mSINGS accuracy at 40 loci was higher with all three cancer types (Supplemental Table S5). In general, mSINGS performed better with fewer loci, MSISensor performed better with more loci, and MANTIS performed well consistently across a broader range of tested loci.

**Figure 4:**
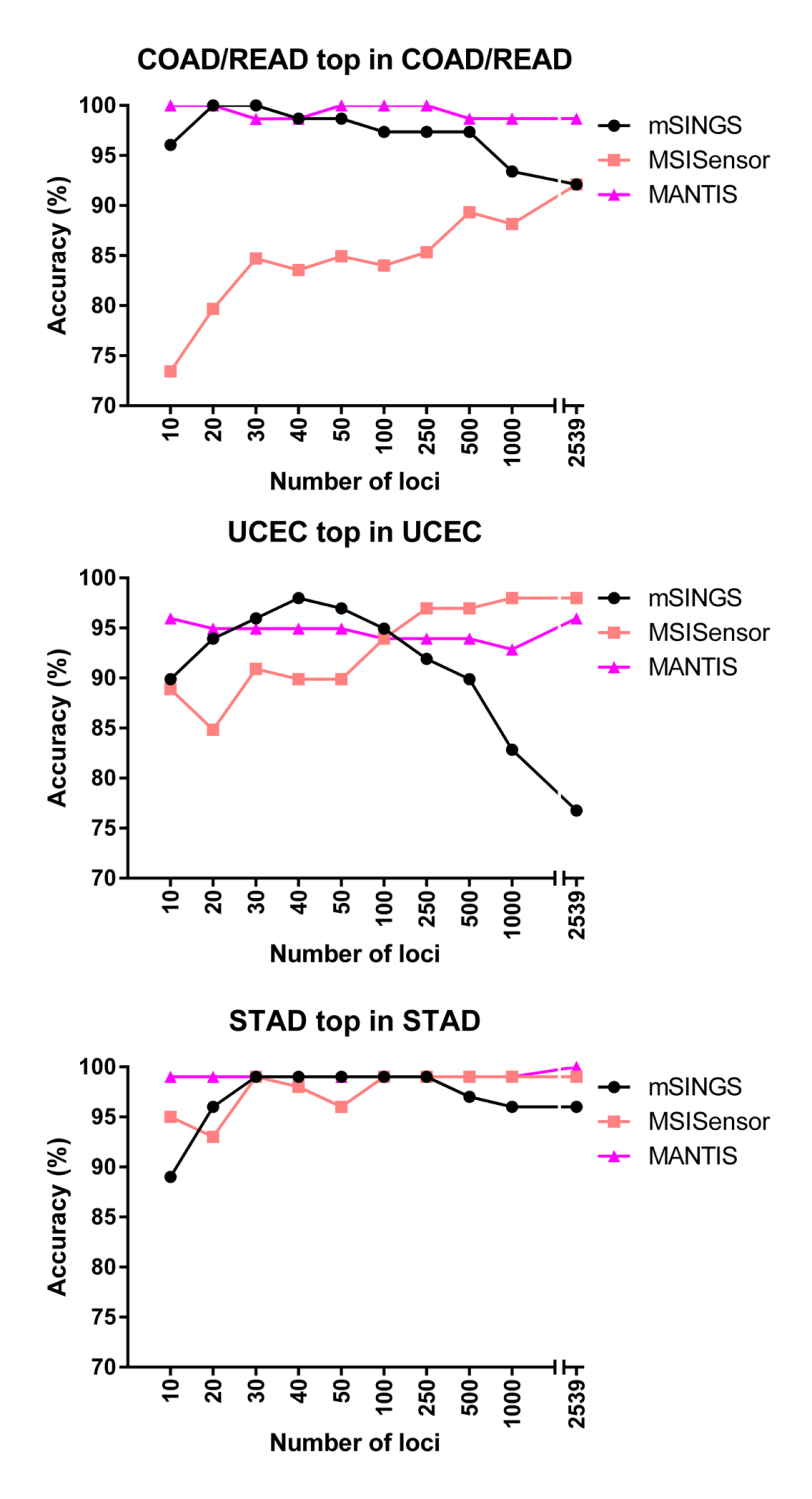
The performance of mSINGS, MSISensor and MANTIS with lists of loci top-performing in COAD/READ, UCEC or STAD For each tool and for each cancer type (COAD/READ, UCEC or STAD), the top-performing loci were determined, and the performance of each tool in each cancer type was evaluated with lists of top loci (particular to only that cancer type) of varying length. The results with 2539 loci (without loci shortlisting) are included for reference. Results are broken down by cancer type.

Previous studies have suggested that different MSI positive cancers may have specific microsatellite loci that are most commonly unstable [10, 11]. For each MSI analysis tool, we sought to account for this by identifying the top-performing loci in each cancer type separately. Unlike in the analysis above, the 10, 20, 30, 40, 50, 100, 250, 500 and 1000 best-performing loci for each tool were determined for each COAD/READ, UCEC and STAD cohort separately (Supplemental File S5). Each tool was then run over each cancer type, with respective top tool-specific and cancer-specific loci (Figure 4). Trends described in the previous analysis remained the same, with performance slightly higher throughout. However in UCEC at 40 loci, mSINGS performed better than MSISensor and MANTIS (98.0% accuracy, *vs*. 89.9% and 94.9% respectively). Also, MANTIS and mSINGS both performed notably better (98.7%) than MSISensor (83.6%) when evaluating COAD/READ samples with 40 loci. The experiments show that the choice of loci being evaluated plays a part in tool performance. While an optimized target panel may allow all tools to perform well, MANTIS exhibits the most stable performance even without such optimizations, providing accurate performance using existing whole-exome data.

## DISCUSSION

We have developed a new tool, MANTIS, for detecting MSI status using paired tumor-normal sequencing data. Unlike other tools, MANTIS analyzes the instability of a normal-tumor sample pair as an aggregate of loci instead of individual loci differences. The approach allows the tool to evaluate the general instability present in a tumor sample, using the data from the corresponding normal sample as an error-correcting baseline. Furthermore, by pooling the scores of all the loci and treating the average as the instability score, the evaluation benefits from the law of large numbers by reducing the impact that sequencing errors or poorly performing loci may have on the results.

We also analyzed the performance of MANTIS, mSINGS, and MSISensor with samples from six cancer types. Overall, MANTIS demonstrated high accuracy across a range of cancer types, and in many cases with restricted sets of well-performing loci. Prior tools have previously been applied to only one of two cancer types, endometrial (MSISensor) and colorectal (mSINGS). With their recommended thresholds, mSINGS and MSISensor are less robust across a range of loci numbers than MANTIS. This appears to be due to the inclusion of poorly performing loci with low sensitivity in the full set of 2539 loci. mSINGS and MSISensor call loci unstable or stable, and MANTIS calculates the instability at each locus. Suboptimal loci may be missed entirely by mSINGS and MSISensor, but still have some increased instability in MSI-H *vs*. MSS samples. Niu et al. recommend a relatively low threshold of 3.5 for MSISensor (*vs*. 20% used by mSINGS and MSI-PCR). This seems to effectively compensate for these poorly performing loci with a large panel, but greatly reduces the specificity of MSISensor when these loci are removed, as in the lists of top-performing loci. Conversely, the threshold of 0.2 (20%) used by mSINGS is effective with a smaller panel of well-performing loci such as the mSINGS authors’ panel MSIplus [11], but this threshold limits the sensitivity of mSINGS with a larger panel (Table 3).

Results from UCEC were least concordant with the above trends. With the full set of 2539 loci, MSISensor performed best, followed closely by MANTIS and distantly by mSINGS. This may potentially arise from differences in the microsatellite instability signatures between COAD/READ and UCEC, the existence of which is supported by previous findings [12, 13]. A recent overview of microsatellite instability across multiple cancer types by Hause et al. further found significant variance in the number of unstable loci present between samples from different diseases [14]. Another potential explanation is that, because mSINGS was developed only with COAD/READ data and MSISensor only with UCEC data, these tools may be overfit to those cancer types. MANTIS, in contrast, was developed and tested using data from multiple cancer types, alleviating the potential overfitting issues that may occur when only including data from a single disease.

Like any NGS-based method, MANTIS performance depends on read coverage, a limitation not shared by MSI-PCR and IHC. Most current clinical guidelines for management of MSI positive tumors are based on a percentage of unstable loci. Current approaches for MSI detection (such as mSINGS, MSISensor, and MSI-PCR) use a discrete fraction of unstable loci to determine to make calls on the status of a sample, but MANTIS provides a continuously valued MSI score that may provide greater utility in determining the level of MSI present in a tumor. Findings by Hause et al. give further credence to this hypothesis, indicating that microsatellite instability may best be viewed as a scaled genotype rather than a simple binary positive or negative classification [14].

We chose MANTIS, mSINGS, and MSISensor for this study since these three tools use NGS to directly assess microsatellite loci in DNA. Other NGS-based methods have been described that indirectly assess MSI through analysis of somatic mutations. MSIseq [15] employs machine learning classification techniques to correlate indels throughout repeat regions to MSI status. Stadler et al. [16] describe a method utilizing a custom NGS assay, and correlating somatic missense and nonsense mutations in protein-coding regions with MSI status. Additionally, Lu et al. [17] describe an algorithm for determining MSI status from RNA-seq data. MSIseq was not included in this study as it is a classifier that only reports MSI-H *vs*. non-MSI-H, without a score or percentage, or information about the instability of particular loci. The Stadler et al. and Lu et al. methods were not included since they cannot be run with the same whole exome sequencing input data, requiring a custom deep-sequenced panel and RNA-seq data, respectively.

Currently, MMR and MSI status are determined in the clinical setting with IHC and MSI-PCR. Conventional multiplex MSI-PCR testing is reported to have 97% sensitivity and 95% specificity [18]. IHC is reported to have 92.4% sensitivity and 99.6% specificity [36]. MSI-PCR with the standard five Bethesda loci is well described for COAD/READ and UCEC [4], but has been shown to perform less accurately in other diseases such as acute myeloid leukemia [10], and may miss MSI in other tumor types. Consideration of only five loci renders conventional MSI-PCR highly susceptible to errors in processing or interpretation of any one locus, and adding additional loci increases cost. IHC is able to effectively determine the presence or absence of the mismatch repair proteins targeted. Unfortunately, IHC cannot detect loss-of-function mutations that do not affect the antigenicity of targeted proteins, or changes to MMR proteins not targeted [2]. Additionally, MSI-PCR and IHC require human interpretation, unlike computational NGS-based methods. Lastly, both MSI-PCR and IHC are clinical laboratory tests that consume fractions of patient tumor samples, unlike computational methods that could be multiplexed with other NGS assays for detecting somatic mutations.

As a tumor *vs*. normal algorithm, MANTIS avoids a time-consuming baseline generation step, eliminates potential baseline bias and allows processing of samples from different sequencing pipelines or tumor types without requiring a different baseline for each. Indeed, sequencing center bias may explain the discordance with mSINGS results from the 4 COAD/READ pairs sequenced at both BCM and BI (Supplemental Table S3). However, matched normal DNA is not always feasibly available for clinical laboratories that only sequence tumor samples, thus a tumor-only method such as mSINGS could reduce both sequencing time and cost to the patient.

The results of this study support several potential directions for future investigation. The accuracy of MANTIS with small numbers of loci suggests that MANTIS could be useful with a targeted sequencing panel designed for MSI testing in the clinic. We have shown that MANTIS performs well in six cancer types; however it (along with other MSI tools) should be further evaluated in a wider variety of cancers. Of particular interest would be evaluation of MANTIS in neurologic, hematologic, pediatric and other malignancies, in which the landscape of MSI is considerably less well described than with COAD/READ and UCEC. With further investigation, incorporating MANTIS into clinical NGS pipelines may permit MSI testing on a large scale, and improve access to emerging therapies that exploit microsatellite instability in cancer.

## MATERIALS AND METHODS

### MANTIS

MANTIS is a tool for identifying microsatellite instability in paired tumor-normal patient samples (Figure 1). It is written as a Python program, utilizing the NumPy (developed with version 1.6.2) and Pysam (developed with version 0.8.3) libraries. Additionally, it requires the reference genome (developed with hg19) [10] in FASTA format to perform alignment of reads spanning the microsatellite loci. The matched normal and tumor inputs are required as indexed BAM files, aligned with any DNA sequence aligner. The targeted loci are required in a 6-column BED file, with the fourth column containing the motif of the microsatellite loci being targeted along with its repeat count in the reference genome, e.g. (AC)12. MANTIS includes a bundled C++ program, RepeatFinder, used to identify microsatellite loci within a reference genome, and create the appropriate BED file. This BED file can be further filtered with BEDTools [20] for regions of interest. Multi-threading is supported and encouraged for larger samples, but is not necessary. More information about the parameters supported by MANTIS is included in the manual available with the software.

Targeted loci are first read from the provided BED file and realigned against the provided reference genome to account for differences between 0- and 1- based indexing. One locus at a time, the tool extracts overlapping reads from the tumor and normal BAM files and performs an initial quality control step to ensure the reads are of sufficient sequence length, meet a minimum average base quality score, and cover the entire targeted locus. Reads passing the initial filtering step are inspected individually to determine the starting position of the microsatellite motif within the read's sequence and the total number of repeats is determined by pattern matching the continuous motif pattern from that starting point. Once the repeat count is determined, a secondary quality control step takes place, ensuring that the locus was not truncated before reaching the end of the read's sequence, and that the microsatellite locus region has a sufficiently high average base quality score. The supporting read count for each of the repeat lengths is determined separately for the tumor and normal files to generate per-locus motif repeat count values.

Once the repeat counts are generated for each locus, a per-locus quality control step takes place. The repeat lengths for both the normal and tumor file are evaluated separately, with values too far from the mean (by default, beyond 3 standard deviations) discarded as outliers. After outliers are removed, each locus is checked for a total number of supporting reads to ensure there is sufficient support to generate a statistically significant distribution for both the normal and tumor files. Loci with substandard coverage are discarded.

The filtered locus repeat count data is then passed to the scoring algorithm that generates an instability score for the sample pair. First, each locus is evaluated separately, with the normal and tumor read distributions normalized to a fraction of each one's total reads, to account for any differences in sequencing depth and coverage. Then, absolute value of the stepwise difference between the tumor and normal distributions is determined:

$$d=\sum_{r\in (R_{r}UR_{N})}\left|T_{r}-N_{r}\right|$$

Where *d* = distance score, *R*T = repeat counts present in tumor, *R*N = repeat counts present in normal, *T*r = normalized read count in tumor supporting repeat of length r, *N*_r_ = normalized read count in normal supporting repeat of length *r*. Once the scores for each locus are assigned, the average of all the locus instability scores is calculated, to provide a single numerical value representing the average aggregate instability present in the sample. Scores reported range from 0.0 (entirely stable) to 2.0 (entirely unstable). The MANTIS software and manual are freely available for download athttps://github.com/OSU-SRLab/MANTIS.

### Comparison of tools

In addition to MANTIS, we tested mSINGS (commit #2e00b6) by Salipante et al. [8] and MSISensor (version 0.2) by Niu et al. [9] (Table 1). mSINGS compares a tumor sample to a pooled normal baseline, generated from the distribution of unique alleles at each microsatellite locus in many normal samples. MSISensor, like MANTIS, compares a tumor sample with its matched normal sample. Both mSINGS and MSISensor determine the stability of each locus analyzed. mSINGS calls a locus unstable if the Z-score of the number of unique alleles at the locus relative to the baseline distribution exceeds a threshold (default: 2). MSISensor calls a locus unstable if a chi-square test of the repeat lengths in the tumor sample *vs*. the normal sample is statistically significant, after Benjamini correction [21]. Both tools then call the sample MSI-H or MSS if the percentage of unstable loci exceeds a threshold.

**Table 1:** Comparison of the MSI-calling tools mSINGS, MSISensor and MANTIS, and the algorithms used by each

Tool	Sample Comparison	Statistical Method	Scoring Approach
mSINGS	Tumor vs. Baseline	Z-score	Per Locus
MSISensor	Tumor vs. Normal	Chi-square	Per Locus
MANTIS	Tumor vs. Normal	Average distance	Aggregate Instability

### Tool parameters

mSINGS was run with Python 2.7.1, VarScan 2.3.6 [22, 23] and SAMtools 0.1.18 [24]. The wrapper script provided with mSINGS was modified (available upon request) to remove its dependency on SCons (http://scons.org/). We generated separate pooled normal baselines from all normal samples within a particular cancer type, according to the mSINGS documentation. The microsatellite loci-containing region BED and .intervals files packaged with mSINGS were used, which contained 2539 loci, as they are appropriate for whole-exome TCGA analyses according to the mSINGS documentation.

MSISensor was slightly modified to output sites that were not called with somatic microsatellite instability along with its other output files (patch files available upon request), and compiled from source. It was run over the test data with the following settings: single-threaded, minimal homopolymer size 1, and minimal microsatellite size 1. All other options were left at their defaults. The microsatellite loci-containing region BED file packaged with mSINGS was used for fairness of comparison, as well as to restrict MSISensor to exomic regions.

MANTIS was run over the test data with three threads. The recommended quality settings for whole-exome data were used, as described in the included MANTIS manual. The microsatellite BED file was derived from the one provided by mSINGS.

### Target loci selection

The 2539 target loci being analyzed were derived from the ones provided with mSINGS and captured in whole exome sequencing datasets, and used with all three tools. Most of the targeted loci (*see* Table 2) were monomer homopolymers of adenine or thymine (95.08%), with only 4.04% of loci being repeats of dimers or longer polymers. This bias towards monomer repeats was expected since intronic mononucleotide repeats outnumber other repeat regions in the human genome [25].

**Table 2:** Breakdown of target loci used for microsatellite status calling. The count and repeat range of each type is listed

Type of Microsatellite	Number of Loci	Min Repeats	Max Repeats	Mean Repeats
Monomer	2436	3	36	15.94
Dimer	96	6	18	14.86
Trimer	4	3	8	4.75
Tetramer	2	7	8	7.5
Pentamer	1	3	3	3.0

**Table 3:** Comparison of accuracy for MSI-H detection

mSINGS				
Metric	COAD/READ	UCEC	COAD/READ + UCEC	STAD
Sensitivity	84.2%	53.1%	66.7%	92.0%
Specificity	100.0%	100.0%	100%	100.0%
Accuracy	92.1%	76.8%	83.4%	96.0%
MSISensor				
Metric	COAD/READ	UCEC	COAD/READ + UCEC	STAD
Sensitivity	92.1%	98.0%	95.4%	98.0%
Specificity	92.1%	98.0%	95.5%	100.0%
Accuracy	92.1%	98.0%	95.4%	99.0%
MANTIS				
Metric	COAD/READ	UCEC	COAD/READ + UCEC	STAD
Sensitivity	100.0%	91.8%	95.4%	100.0%
Specificity	97.4%	100.0%	98.9%	100.0%
Accuracy	98.7%	96.0%	97.1%	100.0%

The performance of mSINGS, MSISensor, and MANTIS over 275 COAD/READ, UCEC, and STAD sample pairs.

### Sample data

In this study, we used data from six cancer types: 76 colon and rectal adenocarcinomas (COAD/READ) [26], 99 uterine corpus endometrial carcinomas (UCEC) [27], 100 gastric adenocarcinomas (STAD) [28], 71 esophageal carcinomas (ESCA), 53 uterine carcinosarcomas (UCS) [29], and 59 prostate adenocarcinomas (PRAD) [30]. We define a “sample” as a single BAM and its accompanying BAI index file, for a tumor or a normal. We define a “pair” as two samples; a tumor and its matched normal, and define a “cohort” as all samples within a cancer type, for a total of 6 cohorts.

Data for all cohorts except PRAD were downloaded from the Cancer Genomics Hub (CGHub), using the CGHub-provided client GeneTorrent [31]. All samples were downloaded in the BAM format, pre-aligned to GRCh37/hg19 [19]. 76 COAD/READ pairs were downloaded, comprised of 38 MSI-H and 38 MSS. COAD/READ data was sequenced at the Baylor College of Medicine (BCM) and the Washington University Genome Sequencing Center (WUGSC) (*Supplemental Table S1B*). 99 UCEC pairs were downloaded, comprised of 49 MSI-H and 50 MSS. All UCEC pairs were sequenced at WUGSC. Next, 100 STAD pairs were downloaded; 50 MSI-H and MSS. All STAD pairs were sequenced at the Broad Institute of MIT and Harvard (BI). The primary authors were blinded to the MSI status of these samples until after initial analysis with mSINGS, MSISensor and MANTIS was completed. Finally, 71 ESCA pairs were downloaded from TCGA; 2 MSI-H and 69 MSS, and 53 UCS pairs were downloaded; 2 MSI-H and 51 MSS. All ESCA and UCS pairs were sequenced at BI. PRAD data was downloaded from dbGaP (accession phs000915.v1.p1) [32] as FASTQ files, using the SRA toolkit [33]. All 59 available PRAD pairs were downloaded; 1 MSI positive and 58 MSI negative (according to the original study for which these samples were sequenced, which only differentiated between MSI positive and MSI negative). PRAD data was sequenced at BI and the University of Michigan (UM) (Supplemental Table S1C). Alignment to hg19 was performed with BWA 0.6.2 [34].

Each of the 916 BAM files (from the 458 tumor-normal pairs in all six cancer types) were sorted and indexed with SAMtools 0.1.18. Deduplication was performed with Picard Tools 1.84 (http://broadinstitute.github.io/picard/), and facilitated with GNU Parallel [35]. The deduplicated BAM files were used for all downstream analyses. Samples used in these analyses are summarized in Supplemental Table S1A, Supplemental File S10.

### Tool performance evaluation

mSINGS, MSISensor and MANTIS were first run on all 175 tumor-normal pairs from COAD/READ and UCEC (Supplemental File S2). A threshold was used for each tool, above which a tumor-normal pair is called MSI positive. For mSINGS, 0.2 (20% of loci called unstable) was used as the threshold for differentiation of MSI positive from MSS predictions, as this is consistent with both MSI-PCR scoring and the threshold used by Salipante et al. A threshold of 3.5% (of loci called unstable) was used for MSISensor, as recommended by Niu et al. For MANTIS, a threshold of 0.4 (average of loci difference scores > 0.4) performed best in testing (see: Results; Relative tool performance), and was used for other analyses. For each tool, the number of true positives, false positives, true negatives, and false negatives was calculated with respect to MSI-PCR status as a gold standard, and this was used to calculate the sensitivity, specificity, error rate, and accuracy of each tool both overall and within each cancer type. Error rate was calculated as (incorrect calls / total calls), and accuracy as (1 - error rate = correct calls / total calls). Note that error rate and accuracy depend on the samples being tested, and cannot be generalized to other data sets, as can sensitivity and specificity. 95% confidence intervals for sensitivity and specificity were calculated using the Wilson score interval with continuity correction [36]. In addition, ranges of thresholds were tested for all three tools in order to determine which thresholds provided optimal performance with the COAD/READ and UCEC test data, allowing comparison of best-case performance. 300 thresholds ranging from 0.001 to 0.3 were tested for mSINGS, 400 thresholds ranging from 0.1% to 40% were tested for MSISensor, and 600 thresholds ranging from 0.001 to 0.6 were tested for MANTIS. After a threshold of 0.4 was chosen for MANTIS, we ran mSINGS, MSISensor and MANTIS over the 100 STAD, 71 ESCA, 53 UCS and 59 PRAD tumor-normal pairs. Tool parameters were selected and performance analyses were performed as described earlier.

For each tool, we measured potential bias arising from differences in sequencing and alignment protocols at different sequencing centers. In addition to the sample data used for tool performance comparison, TCGA data from 4 tumor-normal COAD/READ MSI-H pairs sequenced at both BI and BCM, as well as from 20 tumor-normal UCEC MSI-H pairs sequenced at both BI and WUGSC, was downloaded and preprocessed as earlier (see: *Sample data*) (Supplemental File S3). After deduplication, these pairs were analyzed with mSINGS, MSISensor and MANTIS. MSISensor and MANTIS were run with the parameters described earlier (see: *Tool parameters*). For mSINGS, the normal BAM files were used to generate separate baselines for each cancer type and sequencing center.

In addition to evaluating the statistical performance of each tool, we also evaluated their computational performance (Supplemental Table S2). All three tools were profiled with the runtime and memory usage metrics supplied by the PBS (Portable Batch System) cluster queueing system. MSISensor and MANTIS were tested with one and three threads to evaluate changes in performance (mSINGS does not support multithreading).

### Loci number comparison

For each of the three tools tested, the top-performing 10, 20, 30, 40, 50, 100, 250, 500 and 1000 microsatellite loci within the COAD/READ, UCEC and STAD samples were determined, individually within each cancer type and across all three cohorts. ESCA, UCS and PRAD were not included in this as only five MSI-H cases were available from these cancer types. To determine the top *n* loci for mSINGS and MSISensor, the accuracy of each locus was calculated as if only that locus were to be used to call the MSI status of each tumor sample. For MANTIS, since it calculates instability scores instead of assigning per-locus stability statuses, a difference of averages was calculated for each locus, defined as:

$$a=\frac{\sum_{i\in H}d_{i}}{\left|H\right|}-\frac{\sum_{i\in S}d_{i}}{\left|S\right|}$$

Where *a* = difference of averages, *H* = set of MSI-H samples that cover the locus, *S* = set of MSS samples that cover the locus, and *d*i = distance score of the locus in sample *i*. To compensate for varying locus coverage across samples, this score (accuracy or difference of averages) was then multiplied by the square of the proportion of pairs that had sufficient read coverage at that locus to consider it, as follows:

$$l=ac^{2}$$

Where *l* = locus score, *a* = accuracy or difference of averages, and *c* = proportion of samples that cover the locus. This yielded a performance score for each locus, which allowed all 2539 loci to be ranked. The sensitivity, specificity, error rate, and accuracy of each tool with each loci list was then calculated, both for all three of these cancer types and overall (Supplemental File S4).

### Computing resources

Alignment, deduplication, MSI calling with all three tools, and performance calculations were performed on the Oakley supercomputer at the Ohio Supercomputer Center (https://www.osc.edu/supercomputing/computing/oakley). Figures were generated using GraphPad Prism (version 6.07) and Microsoft^®^ Excel™ 2010. All other performance calculations were performed with custom Perl scripts and Excel™.

## SUPPLEMENTARY MATERIALS FIGURES AND TABLES












