# Supplementary material for: Performance evaluation for rapid detection of pan-cancer microsatellite instability with MANTIS
Source: Oncotarget. 2016 Dec 12;8(5):7452–63. doi: 10.18632/oncotarget.13918 (PMC5352334; doi:10.18632/oncotarget.13918)
Supplement: Supplementary file 1 [file oncotarget-08-7452-s001.pdf]

Performance evaluation for rapid detection of pan-cancer microsatellite instability with MANTIS

Supplementary Material

Supplemental Tables, Figures and Information

Table S1: **A**: Number of samples from each cancer type used for testing and analysis of MSI calling tools. **B**: Breakdown of COAD/READ samples by cancer type and sequencing center. **C**: Breakdown of PRAD samples by sequencing center. WUGSC: Washington University Genome Sequencing Center. BI: Broad Institute of MIT and Harvard. BCM: Baylor College of Medicine. UM: University of Michigan. COAD: Colon Adenocarcinoma. READ: Rectal Adenocarcinoma.

A:

Disease	TCGA Abbreviation(s)	MSI-High (MSI-H)	Microsatellite Stable (MSS)	Total	Sequencing center
Colorectal Adenocarcinoma	COAD/READ	38	38	76	Multiple (see B)
Uterine Corpus Endometrial Carcinoma	UCEC	49	50	99	WUGSC
Gastric Adenocarcinoma	STAD	50	50	100	BI
Esophageal Carcinoma	ESCA	2	69	71	BI
Uterine Carcinosarcoma	UCS	2	51	53	BI
Prostate Adenocarcinoma	PRAD	1	58	59	Multiple (see C)
Combined	–	142	316	458	

B:

TCGA abbreviation	Sequencing Center	MSI-H	MSS	Total
COAD	BCM	33	18	51
COAD	WUGSC	4	9	13
READ	BCM	1	7	8
READ	WUGSC	0	4	4

C:

TCGA abbreviation	Sequencing Center	MSI-H	MSS	Total
PRAD	BI	1	50	51
PRAD	UM	0	8	8

Table S2: Performance profiling of mSINGS, MSISensor and MANTIS over a representative tumor-normal pair (TCGA-V5-A7RE). In **(A)**, the file size of each BAM and total number of reads after deduplication is listed. In **(B)**, runtime, memory usage, and total disk space used by each tool is listed. Runtime is listed as hours:minutes:seconds. mSINGS runtime does not include baseline generation. As MSISensor and MANTIS support multithreading, one thread and three threads were tested for each.

A:

File	File size (bytes)	Total reads
TCGA-V5-A7RE-11A-11D-A351-09.rmdup.bam (normal)	11554546055	82966335
TCGA-V5-A7RE-01A-11D-A351-09.rmdup.bam (tumor)	10689033275	76367232

B:

	mSINGS	MSISensor 1 thread	MSISensor 3 threads	MANTIS 1 thread	MANTIS 3 threads
Runtime	0:26:44	0:04:10	0:04:42	0:02:56	0:02:20
Maximum memory usage	1.48 GB	38 MB	30 MB	89 MB	108 MB
Disk space (intermediate files and results)	32 GB	2.3 MB	2.3 MB	896 KB	896 KB

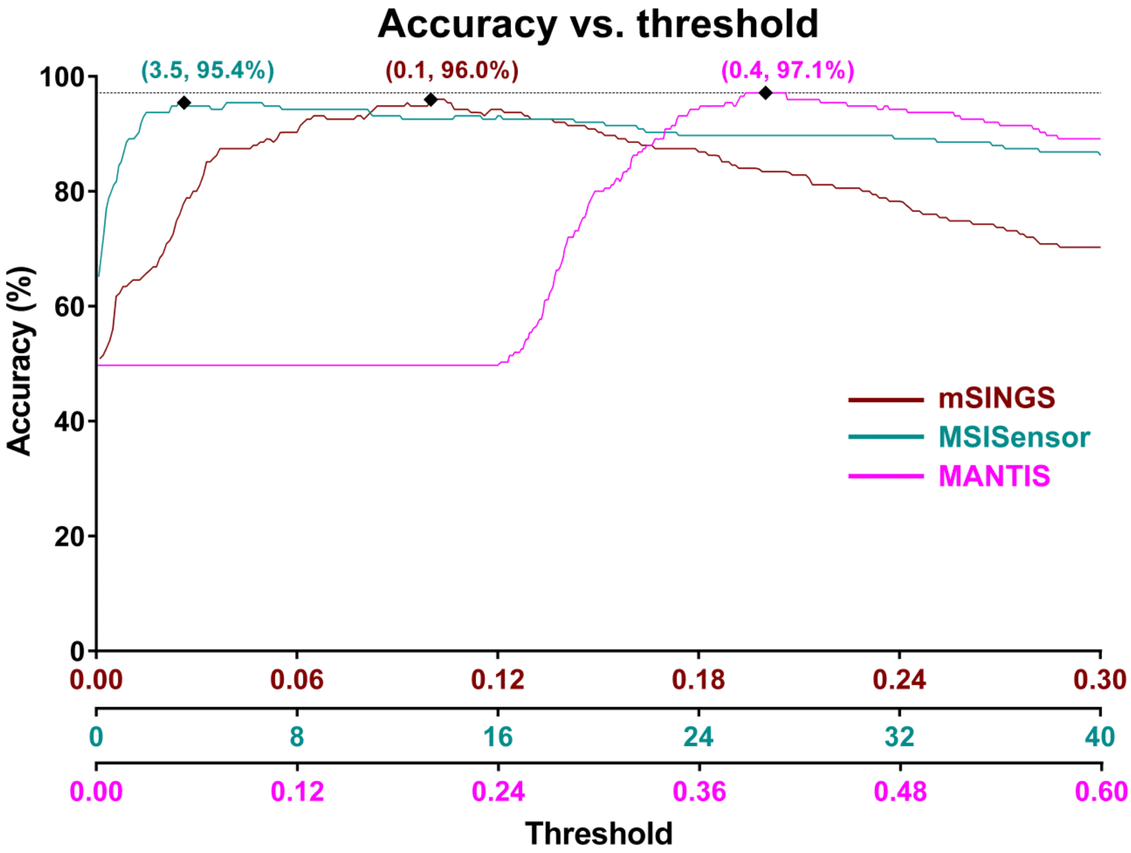

Figure S1: Accuracy of mSINGS, MSISensor and MANTIS at a range of thresholds, when tested with the 76 COAD/READ tumor-normal pairs and 99 UCEC tumor-normal pairs. The thresholds for each tool yielding the highest accuracy for the test data are highlighted. For mSINGS, thresholds from 0.001 to 0.3 were evaluated, in increments of 0.001. For MSISensor, thresholds from 0.1 to 40.0 were evaluated, in increments of 0.1. For MANTIS, thresholds from 0.001 to 0.6 were evaluated, in increments of 0.001.

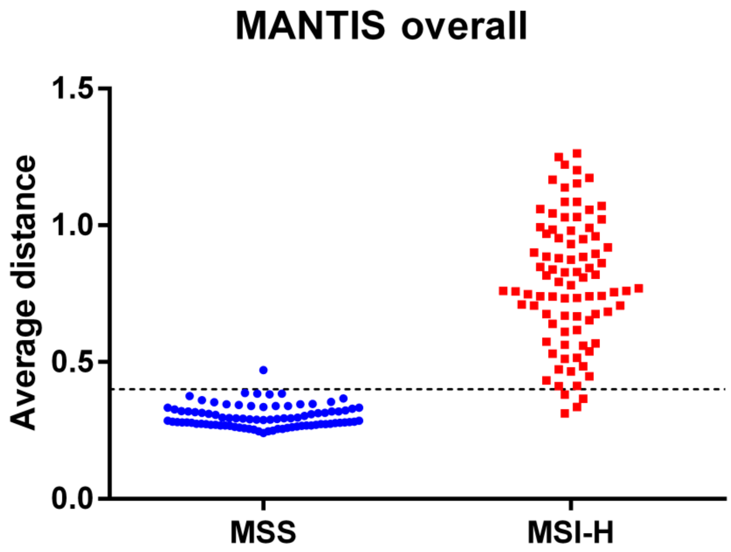

Figure S2: The distribution of MSI scores reported by MANTIS for the 76 COAD/READ tumor-normal pairs and 99 UCEC tumor-normal pairs. The dotted line is at the threshold to call a tumor MSI positive.

A:

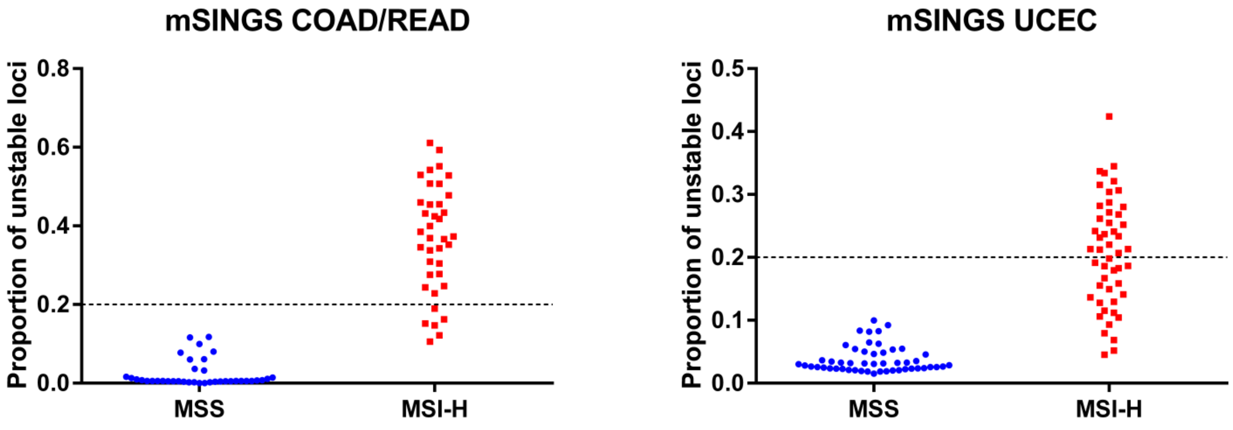

B:

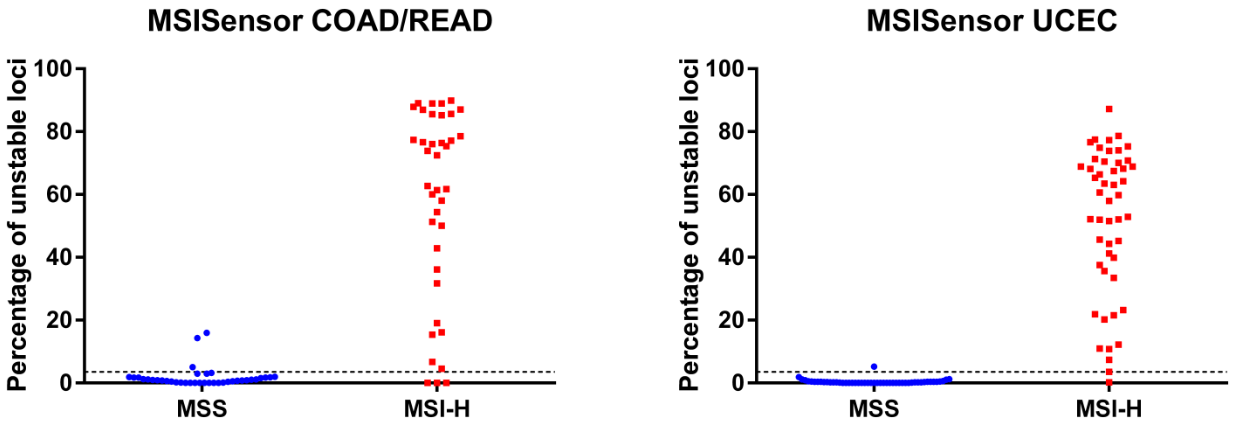

C:

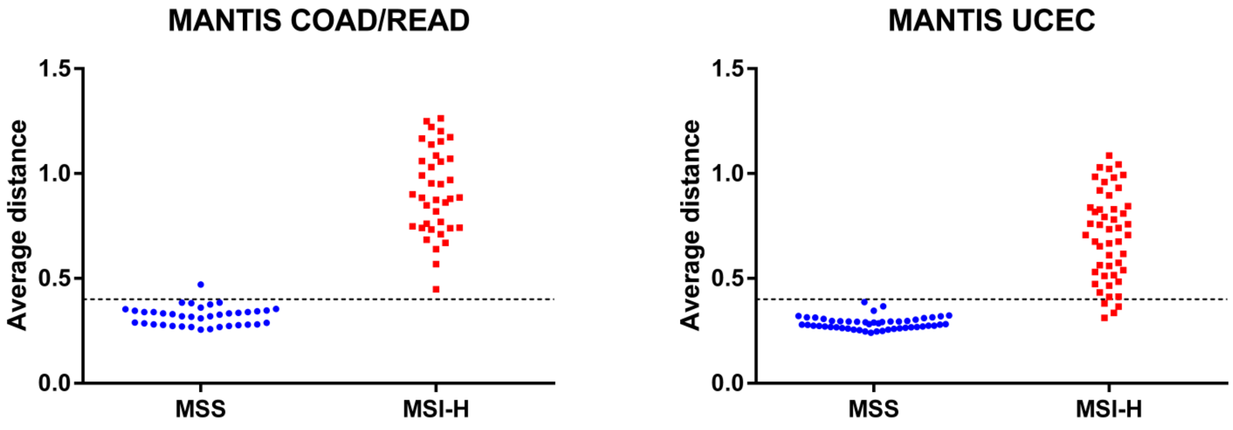

Figure S3: The cumulative distribution of MSI scores reported by mSINGS (**A**), MSISensor (**B**), and MANTIS (**C**) for the 76 COAD/READ tumor-normal pairs and 99 UCEC tumor-normal pairs. The dotted lines are at the tools' respective thresholds to call a tumor MSI positive.

Table S3: The results of mSINGS, MSISensor and MANTIS over a set of 4 COAD/READ MSI-H tumor-normal pairs **(A)** and 20 UCEC MSI-H samples **(B)** that were sequenced at two different sequencing centers. The Pearson correlation coefficient of the tool scores between sequencing centers is provided for each tumor type and tool. (BI: Broad Institute of MIT and Harvard. BCM: Baylor College of Medicine. WUGSC: Washington University Genome Sequencing Center.)

A:

Tool:	mSINGS		MSISensor		MANTIS	
Source:	BI	BCM	BI	BCM	BI	BCM
TCGA-A6-2686	0.5802	0.3924	87.84	87.84	1.173	1.173
TCGA-A6-3809	0.4528	0.5013	77.35	77.69	0.9693	0.9389
TCGA-CK-4951	0.4229	0.2615	61.38	61.38	0.7698	0.7698
TCGA-CM-4746	0.4224	0.4828	62.68	63.3	0.8617	0.8315
R^2	**5.52E-07**		**0.999508**		**0.990615**	

B:

Tool:	mSINGS		MSISensor		MANTIS	
Source:	BI	WUGSC	BI	WUGSC	BI	WUGSC
TCGA-A5-A0GG	0.1809	0.1845	21.95	21.89	0.4113	0.4125
TCGA-AP-A0LS	0.2238	0.2325	52.36	51.98	0.8349	0.8278
TCGA-AP-A1DK	0.1478	0.1437	22.9	23.19	0.5287	0.5307
TCGA-AX-A06H	0.3877	0.3921	59.58	58.99	0.6032	0.6052
TCGA-AX-A1C9	0.099	0.0938	33.07	32.22	0.5654	0.5669
TCGA-AX-A2HD	0.3203	0.3333	70.07	70.04	0.9848	0.9841
TCGA-B5-A11H	0.3792	0.3959	87.2	87.2	0.9698	0.9591
TCGA-BG-A0VW	0.2254	0.2363	51.41	51.57	0.56	0.5592
TCGA-BG-A18B	0.2291	0.2233	66.56	66.56	0.8074	0.8076
TCGA-BS-A0TA	0.3606	0.3695	53.89	54.27	0.5834	0.5831
TCGA-BS-A0V4	0.1381	0.1455	35.16	35.07	0.6156	0.6143
TCGA-D1-A176	0.2303	0.2263	54.85	54.7	0.6719	0.6701
TCGA-D1-A177	0.3162	0.3154	77.22	77.26	1.0279	1.0224
TCGA-D1-A1NS	0.144	0.1502	22.03	21.57	0.5149	0.5162
TCGA-D1-A2G6	0.0696	0.083	0.21	0.21	0.3346	0.3359
TCGA-EC-A24G	0.1105	0.1251	26.8	26.33	0.5147	0.5184
TCGA-EY-A1GF	0.1612	0.1664	51.19	50.95	0.7162	0.7119
TCGA-EY-A1GU	0.2328	0.2305	61	60.66	0.9327	0.9318
TCGA-EY-A1H0	0.3288	0.3239	67.35	67.39	0.9545	0.9473
TCGA-EY-A215	0.308	0.3043	59.49	58.84	0.8958	0.8974
R^2	**0.994194**		**0.999791**		**0.999808**	

Table S4: The performance of mSINGS, MSISensor, and MANTIS over all 458 pairs from COAD/READ, UCEC, STAD, ESCA, UCS and PRAD.

	**mSINGS**						
	**COAD/READ**	**UCEC**	**STAD**	**ESCA**	**UCS**	**PRAD**	**Overall**
Threshold:	0.2	0.2	0.2	0.2	0.2	0.2	0.2
TP:	32	26	46	2	1	1	108
FP:	0	0	0	0	0	1	1
TN:	38	50	50	69	51	57	315
FN:	6	23	4	0	1	0	34
**Sensitivity:**	**84.21%**	**53.06%**	**92.00%**	**100.00%**	**50.00%**	**100.00%**	**76.06%**
Lower bound (95% CI):	68.07%	38.42%	79.89%	19.79%	2.67%	5.46%	68.03%
Upper bound (95% CI):	93.41%	67.22%	97.41%	100.00%	97.33%	100.00%	82.64%
**Specificity:**	**100.00%**	**100.00%**	**100.00%**	**100.00%**	**100.00%**	**98.28%**	**99.68%**
Lower bound (95% CI):	88.57%	91.11%	91.11%	93.43%	91.27%	89.54%	97.97%
Upper bound (95% CI):	100.00%	100.00%	100.00%	100.00%	100.00%	99.91%	99.98%
**Error rate:**	**7.89%**	**23.23%**	**4.00%**	**0.00%**	**1.89%**	**1.69%**	**7.64%**
**Accuracy:**	**92.11%**	**76.77%**	**96.00%**	**100.00%**	**98.11%**	**98.31%**	**92.36%**

	**MSISensor**						
	**COAD/READ**	**UCEC**	**STAD**	**ESCA**	**UCS**	**PRAD**	**Overall**
Threshold:	3.5	3.5	3.5	3.5	3.5	3.5	3.5
TP:	35	48	49	2	2	1	137
FP:	3	1	0	0	0	0	4
TN:	35	49	50	69	51	58	312
FN:	3	1	1	0	0	0	5
**Sensitivity:**	**92.11%**	**97.96%**	**98.00%**	**100.00%**	**100.00%**	**100.00%**	**96.48%**
Lower bound (95% CI):	77.52%	87.76%	87.99%	19.79%	19.79%	5.46%	91.55%
Upper bound (95% CI):	97.94%	99.89%	99.90%	100.00%	100.00%	100.00%	98.70%
**Specificity:**	**92.11%**	**98.00%**	**100.00%**	**100.00%**	**100.00%**	**100.00%**	**98.73%**
Lower bound (95% CI):	77.52%	87.99%	91.11%	93.43%	91.27%	92.26%	96.57%
Upper bound (95% CI):	97.94%	99.90%	100.00%	100.00%	100.00%	100.00%	99.59%
**Error rate:**	**7.89%**	**2.02%**	**1.00%**	**0.00%**	**0.00%**	**0.00%**	**1.97%**
**Accuracy:**	**92.11%**	**97.98%**	**99.00%**	**100.00%**	**100.00%**	**100.00%**	**98.03%**

	MANTIS						
	**COAD/READ**	**UCEC**	**STAD**	**ESCA**	**UCS**	**PRAD**	**Overall**
Threshold:	0.4	0.4	0.4	0.4	0.4	0.4	0.4
TP:	38	45	50	2	2	1	138
FP:	1	0	0	0	0	0	1
TN:	37	50	50	69	51	58	315
FN:	0	4	0	0	0	0	4
**Sensitivity:**	**100.00%**	**91.84%**	**100.00%**	**100.00%**	**100.00%**	**100.00%**	**97.18%**
Lower bound (95% CI):	88.57%	79.52%	91.11%	19.79%	19.79%	5.46%	92.50%
Upper bound (95% CI):	100.00%	97.35%	100.00%	100.00%	100.00%	100.00%	99.09%
**Specificity:**	**97.37%**	**100.00%**	**100.00%**	**100.00%**	**100.00%**	**100.00%**	**99.68%**
Lower bound (95% CI):	84.57%	91.11%	91.11%	93.43%	91.27%	92.26%	97.97%
Upper bound (95% CI):	99.86%	100.00%	100.00%	100.00%	100.00%	100.00%	99.98%
**Error rate:**	**1.32%**	**4.04%**	**0.00%**	**0.00%**	**0.00%**	**0.00%**	**1.09%**
**Accuracy:**	**98.68%**	**95.96%**	**100.00%**	**100.00%**	**100.00%**	**100.00%**	**98.91%**

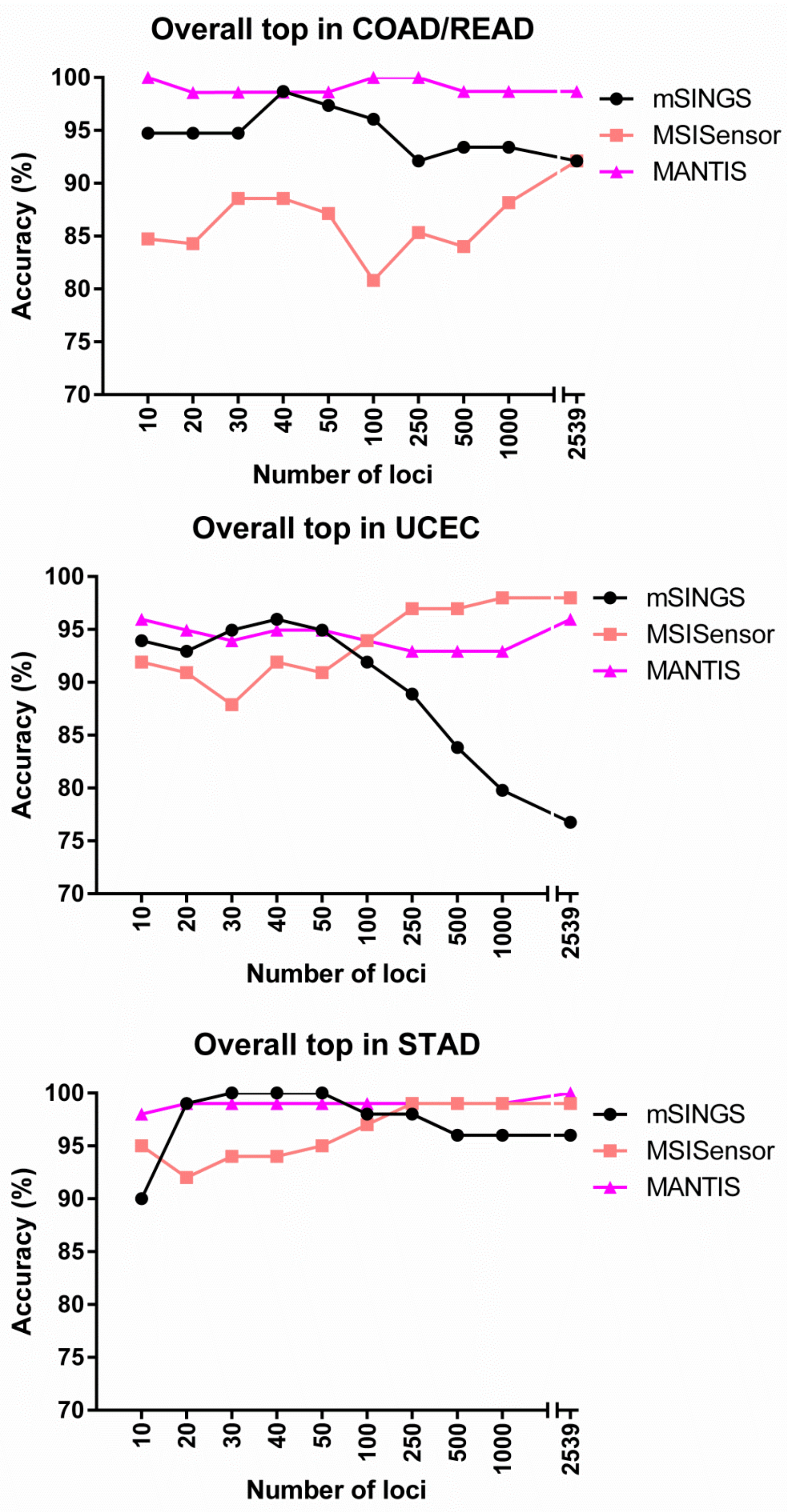

Figure S4: The performance of mSINGS, MSISensor and MANTIS with lists of top-performing loci. For each tool, and within the COAD/READ, UCEC and STAD samples, the top-performing loci were determined, and the performance of each tool was evaluated with lists of top loci of varying length. The results with 2539 loci (without loci shortlisting) are included for reference. Results are broken down by cancer type.

Table S5: Loci number comparison. The performance of mSINGS, MSISensor and MANTIS with their lists of top loci in COAD/READ, UCEC and STAD**.** The accuracy of each tool over the 275 tumor-normal pairs of test data (in COAD/READ, UCEC and STAD) with each loci list is provided. Top loci were determined using the samples from all three cancer types. The results with 2539 loci (without loci shortlisting) are included for reference and are broken down per-cancer type.

		Number of loci:									
**COAD/READ**		10	20	30	40	50	100	250	500	1000	2539
	mSINGS	94.74%	94.74%	94.74%	98.68%	97.37%	96.05%	92.11%	93.42%	93.42%	92.11%
Tool:	MSISensor	84.75%	84.29%	88.57%	88.57%	87.14%	80.82%	85.33%	84.00%	88.16%	92.11%
	MANTIS	100.00%	98.59%	98.61%	98.61%	98.63%	100.00%	100.00%	98.68%	98.68%	98.68%

		Number of loci:									
**UCEC**		10	20	30	40	50	100	250	500	1000	2539
	mSINGS	93.94%	92.93%	94.95%	95.96%	94.95%	91.92%	88.89%	83.84%	79.80%	76.77%
Tool:	MSISensor	91.92%	90.91%	87.88%	91.92%	90.91%	93.94%	96.97%	96.97%	97.98%	97.98%
	MANTIS	95.96%	94.95%	93.94%	94.95%	94.95%	93.94%	92.93%	92.93%	92.93%	95.96%

		Number of loci:									
**STAD**		10	20	30	40	50	100	250	500	1000	2539
	mSINGS	90.00%	99.00%	100.00%	100.00%	100.00%	98.00%	98.00%	96.00%	96.00%	96.00%
Tool:	MSISensor	95.00%	92.00%	94.00%	94.00%	95.00%	97.00%	99.00%	99.00%	99.00%	99.00%
	MANTIS	98.00%	99.00%	99.00%	99.00%	99.00%	99.00%	99.00%	99.00%	99.00%	100.00%

Supplemental Files:

- S1: Summary of the TCGA (The Cancer Genome Atlas) and SU2C (Stand Up To Cancer) data used for main comparisons.
- S2: mSINGS, MSISensor and MANTIS scores for all 458 pairs used for testing and analysis of MSI calling tools. The results from the 24 pairs used for sequencing center comparison (see Supplemental Table S5) are not included.
- S3: Summary of the TCGA data used for tool comparisons by sequencing center.
- S4: The performance of each locus assessed with mSINGS, MSISensor and MANTIS, within COAD/READ, UCEC, STAD, and all three cancer types taken together. Note that locus performance scores from MANTIS are not directly comparable to those from mSINGS or MSISensor, due to the different algorithms used by each.
- S5: Performance of mSINGS, MSISensor and MANTIS in COAD/READ, UCEC and STAD, with each list of top loci (10, 20, 30, 40, 50, 100, 250, 500 or 1000, from COAD/READ, UCEC, STAD or overall, in COAD/READ, UCEC, STAD or overall, with mSINGS, MSISensor or MANTIS).
